# Management and climate contributions to satellite-derived active fire trends in the contiguous United States

**DOI:** 10.1002/2013JG002382

**Published:** 2014-04-28

**Authors:** Hsiao-Wen Lin, Jessica L McCarty, Dongdong Wang, Brendan M Rogers, Douglas C Morton, G James Collatz, Yufang Jin, James T Randerson

**Affiliations:** 1 Department of Earth System Science, University of CaliforniaIrvine, California, USA; 2 Michigan Tech Research InstituteAnn Arbor, Michigan, USA; 3 Department of Geographical Sciences, University of MarylandCollege Park, Maryland, USA; 4 Biospheric Sciences Laboratory, NASA Goddard Space Flight CenterGreenbelt, Maryland, USA

## Abstract

Fires in croplands, plantations, and rangelands contribute significantly to fire emissions in the United States, yet are often overshadowed by wildland fires in efforts to develop inventories or estimate responses to climate change. Here we quantified decadal trends, interannual variability, and seasonality of Terra Moderate Resolution Imaging Spectroradiometer (MODIS) observations of active fires (thermal anomalies) as a function of management type in the contiguous U.S. during 2001–2010. We used the Monitoring Trends in Burn Severity database to identify active fires within the perimeter of large wildland fires and land cover maps to identify active fires in croplands. A third class of fires defined as prescribed/other included all residual satellite active fire detections. Large wildland fires were the most variable of all three fire types and had no significant annual trend in the contiguous U.S. during 2001–2010. Active fires in croplands, in contrast, increased at a rate of 3.4% per year. Cropland and prescribed/other fire types combined were responsible for 77% of the total active fire detections within the U.S and were most abundant in the south and southeast. In the west, cropland active fires decreased at a rate of 5.9% per year, likely in response to intensive air quality policies. Potential evaporation was a dominant regulator of the interannual variability of large wildland fires, but had a weaker influence on the other two fire types. Our analysis suggests it may be possible to modify landscape fire emissions within the U.S. by influencing the way fires are used in managed ecosystems.

**Key Points:**

1. Wildland, cropland, and prescribed fires had different trends and patterns
2. Sensitivity to climate varied with fire type
3. Intensity of air quality regulation influenced cropland burning trends

## 1. Introduction

For over a century, considerable effort has been invested in systematically monitoring and managing fires in the United States, with the aim of minimizing threats to human health and property and maintaining ecosystem function and biodiversity [*Pyne*, 2001; *Stephens and Ruth*, 2005]. Although fires are now widely used as a tool in land management, they also had an important role in regulating ecosystem processes prior to extensive human settlement [*Marlon et al.*, 2008; *Bowman et al.*, 2009]. Climate influences fire on multiple time scales, including by determining distributions of plant functional types and species over a period of decades to centuries [*van der Werf et al*., 2008], the amount and characteristics of lightning and other ignition sources [*Rorig and Ferguson*, 2002], fuel loads and moisture [*Collins et al.*, 2006], the length of the fire season, and fire weather during individual events [*Bessie and Johnson*, 1995]. Fires, in turn, modify climate through aerosol and greenhouse gas emissions [*Crutzen and Andreae*, 1990; *Langenfelds et al.*, 2002; *Kasischke et al.*, 2005] and by changing land surface properties [*Amiro et al.*, 2006; *Lyons et al.*, 2008; *Lee et al.*, 2011; *Jin et al.*, 2012]. These two way climate-fire interactions create the potential for regional and global scale feedbacks, with their magnitude and sign likely varying regionally [e.g., *Rogers et al.*, 2013; *Tosca et al.*, 2013]. With fires playing an important role in modifying many Earth system and ecosystem processes, an important challenge is to understand the role of management and climate in controlling contemporary changes in fire activity.

Multiple fire regimes exist within the U.S. as a consequence of considerable regional variability in ecosystems, climate, and land use. Large wildfires in western U.S. forests and shrublands are dominant contributors to regional and contiguous U.S. (CONUS) burned area [*Littell et al.*, 2009; *Kasischke et al.*, 2011]. For this class of fires, interannual and decadal-scale variability is often driven by fire weather controls during summer and by cumulative winter precipitation levels over several years that influence the continuity of surface fuels [*Swetnam and Betancourt*, 1990; *Westerling et al.*, 2003; *Crimmins*, 2006]. The use of prescribed fire in forests as a federal policy started in the 1960s, when studies showed that landscape-level changes in ecosystem composition could be attributed to fire suppression [*Stephens and Ruth*, 2005]. In cropland areas, fire is frequently applied to clear fields of crop residues and to manage pests and disease [e.g., *Smiley et al.*, 1996]. As a consequence, cropland fires vary considerably among different crop types [*McCarty et al.*, 2007, 2009; *Tulbure et al.*, 2010; *Lin et al.*, 2012]. Prairies of the Great Plains are burned every 1–2 years to avoid woody encroachment and enhance grazing productivity [*Brockway et al.*, 2002; *Simmons et al.*, 2007; *Allen and Palmer*, 2011]. Fires are also used for ecosystem management in pine forests in the southeast; prescribed fires, usually at 2–5 year intervals, are used to prepare the site before seeding and planting, to remove logging debris, and to manage understory species [*Waldrop et al.*, 1992; *Carter and Foster*, 2004].

Recent changes in climate have contributed to increases in the number of large wildfires in North America, as a consequence of longer fire seasons and warmer, drier conditions during summer [*Gillett et al.*, 2004; *Kasischke and Turetsky*, 2006; *Westerling et al.*, 2006; *Morton et al.*, 2013]. With expected changes in climate over the next several decades, burned area for wildfires will likely increase [*Spracklen et al.*, 2009], with individual fires becoming more intense and severe [*Westerling et al.*, 2011]. However, much less is known about the climate sensitivity of cropland, rangeland, and plantation fires, or the underlying mechanisms regulating these sensitivities. As a result, it is difficult to estimate how the contributions of managed fires to regional and continental-scale emissions will evolve in the future. For example, while there is evidence showing that drought events decrease the occurrence of cropland fires in Australia [*Lin et al.*, 2012], it remains unclear if this is caused by drought-induced reductions in crop yields and thus fuel loads, or from farmers igniting fewer fires during warmer and drier periods. This is an example of a critical gap in our understanding, and one that can be partly addressed by combining analysis of satellite imagery with other spatially distributed agricultural data sets.

Satellite observations of burned area and active fires (thermal anomalies) provide consistent and systematic coverage, which enables assessment of changes in cropland and prescribed fires at a continental scale [*Korontzi et al.*, 2006; *McCarty et al.*, 2009]. Satellite data are suitable for evaluating fire impacts on air quality or surface characteristics [*Chu et al.*, 2003; *Engel-Cox et al.*, 2004; *Liu et al.*, 2005; *Park et al.*, 2007; *Beck et al.*, 2011] and complement existing state and federal reports that typically document fire statistics using the number of occurrences or area burned within a given region. While existing state and federal reporting systems provide valuable information for many wildland fires and some prescribed fires, cropland burning has not yet been specifically targeted, and thus emissions and trends for this fire type remain highly uncertain. Given this limitation, satellite observations that provide comprehensive coverage of the U.S. have the potential to improve our understanding of the relative importance of different fire types and their relationship with environmental drivers. However, an understanding of the characteristics of each satellite sensor, including spectrometer sensitivities and orbit characteristics, is needed to properly interpret these observations.

Two basic approaches exist for sampling fire patterns and trends with remote sensing data. The first is to map burned area using surface reflectance imagery from pre- and post-burn periods [*Tansey et al.*, 2004; *Giglio et al.*, 2009]. The second is to quantify actively burning fire fronts using measurements of surface thermal anomalies [*Giglio et al.*, 2003a; *Arino et al.*, 2005; *Roberts et al.*, 2011]. Both remote sensing data streams have strengths and weaknesses with respect to their use in quantifying fire trends at a continental scale. Burned area more immediately lends itself to computing emissions, if it can be combined with information on fuel loads and combustion completeness [*Seiler and Crutzen*, 1980]. However, most global-scale remote sensing products of burned area are derived from surface reflectance imagery with moderate resolution (∼500 m to 1 km) [e.g., *Roy et al.*, 2005]. This resolution is suitable for mapping large wildfires in savannas and boreal forests but can be insufficient for tracking fires that are much smaller than the spatial resolution of an individual surface reflectance pixel—as is often the case for cropland or plantation fires and many prescribed fires [*McCarty*, 2011; *Randerson et al.*, 2012]. Surface reflectance-based products with a higher spatial resolution, such as Landsat with a 30 m pixel size, have the advantage of capturing pre- and post-fire surface reflectance changes suitable for landscape level analysis [e.g., *Epting and Verbyla*, 2005; *Eidenshink et al.*, 2007]. However, the temporal resolution of Landsat with a 16 day repeat cycle may not be suitable for capturing many cropland or plantation fires that last for a short period and are often immediately followed by other forms of land management such as plowing or harvesting. Here we chose to use the MODIS active fire (thermal anomaly) product for our analysis at a continental level, because this product can detect fire fronts that are an order of magnitude smaller than moderate resolution burned area products [*Giglio et al.*, 2003b]. The higher resolution of the active fire detections is important for systematically quantifying long-term trends in agricultural and prescribed fires and for quantitatively comparing activity levels across different fire types.

In this paper, our goal was to quantify trends in satellite-derived time series of active fires as a function of fire type. The satellite observations we used from MODIS provide a statistical sampling (“daily snapshot”) of the distribution of fire thermal anomalies across the landscape. We divided these MODIS observations for the contiguous U.S. into three classes according to fire type based on a combination of remote sensing burned area and land cover products. The three fire types were large wildland fires, cropland fires, and prescribed/other fires. Although past work provides evidence for strong climate control on wildfires, less is known about relationships with climate for cropland and prescribed/other fires. We hypothesized that climate control of the latter two fire types was weaker than for large wildland fires, as a result of land managers regulating patterns of ignition. Our analysis identified differences in the response of several fire types to climate, thus providing information that is needed for the design of efficient fire management policies that account for projected climate changes during the next few decades. Finally, for cropland fires, we analyzed how long-term trends may respond to differences in the intensity of air quality policies enacted by different states. We hypothesized that states with stronger air quality regulation would have smaller increases (or greater reductions) in cropland fires over the past decade compared to states with fewer controls.

## 2. Data Sets and Methods

### 2.1. MODIS Active Fire Detection Products

We used MODIS Global Fire Location Product (MCD14ML) collection 5 for the observation of actively burning fires [*Giglio et al.*, 2006]. This product reports the daily latitude and longitude location information of actively burning fires at a spatial resolution of 1 km, along with quality flags, scan angle information, and fire radiative power. We used all of the Terra active fires in this time series in our analysis, including detections with low, medium, and high confidence levels. The MODIS sensors, with dedicated 3.9 and 11.0 µm fire channels, have equator overpass times at approximately 10:30 am and 10:30 pm local time for Terra and 1:30 pm and 1:30 am local time for Aqua [*Giglio et al.*, 2006]. Here we used Terra data set for its longer time span (2001–2010 in this study), although Aqua generally detects more active fires because the overpass time is closer to mid-day and more fires occur at this time. Static sources (i.e., gas flares or industrial heat sources) were removed prior to analysis by excluding any detection that was near the coordinates of an existing hot spot library [*Giglio et al.*, 2006].

Using these active fire detections, we were able to statistically sample the number of thermal anomaly hot spots associated with different types of fires at the time of the satellite overpass. Given the polar orbit of the Terra satellite, this sampling approach yielded many separate active detections within the perimeter of large wildland fires because these fires often lasted for many days, had more spatially extensive flaming fronts, and thus were scanned multiple times by MODIS, which images each location within the U.S. with a repeat time of 1–2 days. Similarly, smaller and shorter-lived fires were sampled less frequently. As such, this sampling approach provided a statistically robust snapshot view of fire activity across the landscape but not a comprehensive inventory of all fires. Past work indicates that there is often a close linear relationship fire radiative energy and fire emissions [e.g., *Roberts et al.*, 2005; *Wooster et al.*, 2005; *Vermote et al.*, 2009; *Xu et al.*, 2010]. Across different regions, variation in the number of active fire detections contributes substantially to variation in fire radiative energy [*Roberts et al.*, 2011], and thus the active fire data presented here contain some information about the relative pattern of fire emissions.

### 2.2. Monitoring Trends in Burn Severity (MTBS) Data

**Figure 1:**
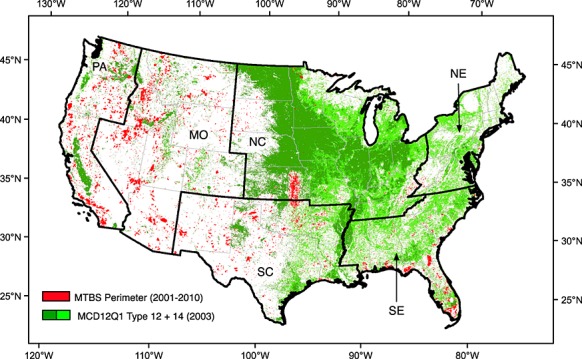
Large wildland fire and cropland masks used for fire type attribution. We used Monitoring Trends in Burn Severity (MTBS) fire perimeters from years 2001–2010 to identify large wildland fires. For croplands, we used the IGBP permanent cropland (type 12; shown in dark green) and cropland and natural vegetation mixed class (type 14; shown in light green) from the MODIS MCD12Q1 product. For simplicity, here we only show the cropland map for 2003. The contiguous US was divided into six regions: the Pacific (PA), mountain (MO), northcentral (NC), northeast (NE), southcentral (SC), and southeast (SE), to assess regional trends and climate sensitivities.

We used the MTBS data to identify the perimeter of large wildland fires. The MTBS project (Figure 1) is a joint effort between the U.S. Department of Agriculture Forest Service and the U.S. Geological Survey to map large fires from 1984 to the present. The MTBS reports burn severity and the perimeter of large fires using Landsat observations with a 30 m spatial resolution [*Eidenshink et al.*, 2007]. Large fires are defined as those greater than 1000 acres (405 ha) in the west and 500 acres (202 ha) in the east of the contiguous United States. The east-west dividing line for this difference in measurement protocol is along the eastern border of North Dakota, South Dakota, Nebraska, Kansas, Oklahoma, and Texas. The MTBS data set includes large fires from all land ownership classes and three different fire types, including wildfires, wildland fire use (i.e., use of naturally occurring wildland fires for pre-planned land management objectives), and prescribed fires. For the 2001–2010 period, wildland fire use and prescribed fires accounted for approximately 5% of the total fire perimeter area. For our analysis, we converted the geographic information system (GIS) vector polygons from the National MTBS Burned Area Boundaries Dataset (downloaded from www.mtbs.gov in June 2012) to an annual raster mask with a 1 km resolution to match other data sets, based on the criteria that at least 50% of the 1 km cell had to occur within the MTBS perimeter. Prior to converting polygons into raster images, we also applied a 1 km buffer around every perimeter reported by MTBS. This was done to assign active fires near the edge of MTBS perimeters to the large wildland fire class, given uncertainties in the spatial location of MODIS active fires.

### 2.3. Cropland Data

The MODIS Land Cover Type Product (MCD12Q1) Collection 5 (C5) provides multiple land type classification systems with spatial resolution of 500 m and annual coverage from 2001 to 2009 [*Friedl et al.*, 2002]. For our cropland layer, we used the International Geosphere Biosphere Program classes for cropland (class 12) and cropland-natural vegetation mixture (class 14) vegetation types from MODIS tiles H8V4 through H12V6, which cover the contiguous United States (Figure 1). To attribute active fires to croplands, we created a new 1 km crop mask that was labeled as crop if any of the four 500 m grid cells in the original MCD12Q1 product were from classes 12 or 14. During 2001–2009, we used the annual land cover type product for each individual year. We also reused the 2009 product for fire attribution in year 2010, since Collection 5 stopped at 2009 and a software issue existed for cropland mapping in Collection 5.1.

### 2.4. Fire Type Attribution

**Figure 2:**
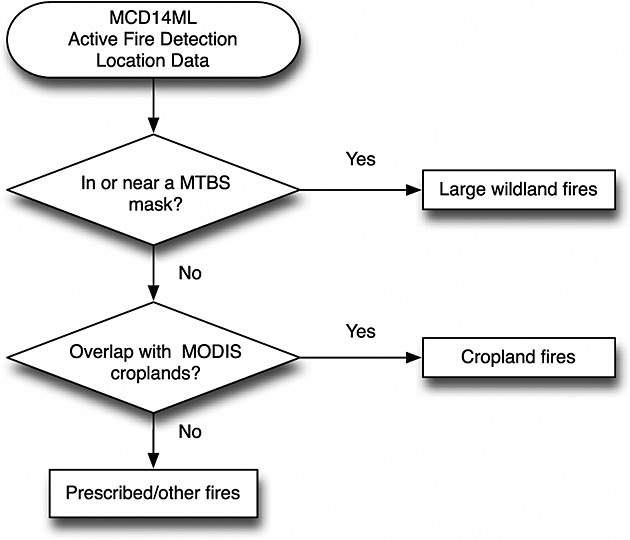
Workflow for the attribution of active fires detected by MODIS (MCD14ML) into the three fire types defined in this study.

We attributed the total number of active fire detections to individual fire types using a step-by-step workflow (Figure 2). We first evaluated whether the latitude and longitude coordinates of active fire detections were within the perimeter of the MTBS mask and classified these hot spots as being associated with large wildland fires. The MTBS data set did not exclude prescribed or wildland fire use perimeters as long as burned area exceeded a minimum threshold, although these components were small component of the total burned area. When the active fire detection coordinates were outside of the MTBS mask, we repeated the process with the MCD12Q1 cropland mask and classified this set as being associated with cropland burning. Residual active fire detections that overlapped with neither MTBS nor cropland masks were defined as fire detections associated with prescribed or other fires (hereafter referred to as prescribed/other). The prescribed/other class included prescribed burns that were smaller than MTBS minimum size thresholds. In many areas, these smaller prescribed fires were likely the dominant contributor to the total burned area of prescribed fires. The prescribed/other class also likely included a small number of active fire detections associated with wildland fires that were smaller than the MTBS minimum size thresholds as well as agricultural fires in areas where the croplands were not detected by the MODIS land cover product.

The attribution process was performed at a 1 km resolution and aggregated into half-degree (0.5°) grid cells for further mapping and analysis. The 0.5° products were then adjusted with a latitude-dependent scalar [*Giglio et al.*, 2003b] to account for latitudinal differences in the spatial-temporal coverage of the polar orbiting Terra satellite. In addition, there was a missing period for Terra active fires from 16 June to 2 July 2001. We corrected for this gap by multiplying remaining active fires in June and July 2001 by a ratio consisting of the number of days in a full month and the number of days with valid observations within each respective month.

### 2.5. Climate Data

We used a monthly potential evaporation (PE) data set derived by *Morton et al.* [2013] as an indicator of fuel moisture conditions and the potential for fire spread. The data set was calculated from the Food and Agriculture Organization (FAO) version of the Penman Monteith equation [*Allen et al.*, 1998] from saturation vapor pressure, net radiation, ground heat flux, and vapor pressure deficit. Climate variables necessary for the equation were obtained from the National Centers for Environmental Prediction (NCEP) North American Regional Reanalysis at a 3 h time step [*Mesinger et al.*, 2006]. PE was subsequently averaged into a monthly time series. We chose PE to represent climate impacts on fires because of the strong relationship between the interannual variability of burned area and PE in North America documented in past work [*Morton et al.*, 2013] and because PE integrates known drivers of fire weather.

### 2.6. Statistical Analysis for Assessing Climate-Fire Relationships

We performed linear regression and calculated correlation coefficients between PE and each fire type to test our hypothesis that weaker climate controls existed for cropland and prescribed/other fire types. Using a climatology of monthly mean active fire detections derived from 2001–2010 observations, we defined the peak fire season at each grid cell as the three consecutive months that accounted for the largest number of detections and also included the month with the maximum number of detections. For each fire type, we then computed linear regressions between PE and the sum of active fire detections during the fire season. Comparison between active fire detections and PE were made either for 5° grid cells or for CONUS subregions (Figure 1). PE for 5° grid cells or subregions was calculated by constructing an area-weighted average from the original 0.5° data set.

### 2.7. Study Area

#### 2.7.1. Regions for Primary Analysis

To assess regional trends and sensitivity to climate variability, we divided the contiguous U.S. into six major geographical regions: Pacific (PA), Mountain (MO), Northcentral (NC), Northeast (NE), Southcentral (SC), and Southeast (SE) (Figure 1). The regions were assigned by states, roughly following the National Air Quality Control Regions from the National Interagency Coordination Center (http://www.predictiveservices.nifc.gov/NPSG/NPSS.htm).

#### 2.7.2. Assessing Cropland Active Fire Trends as a Function of the Intensity of Fire Management Policy

We aggregated cropland fires in different states into fire policy classes to test if states with stricter air quality policies and fire regulation systems had smaller long-term increases in cropland fires compared to states with fewer controls. We systematically investigated differences in fire policy within different states and decided upon four classes with increasing levels of intervention among 22 states that had open burning policies: (1) states with open burning or prescribed fire laws, but where there was no required reporting to state agencies, (2) states with open burning or prescribed fire laws, and where a state agency permit was required prior to ignition, (3) in addition to existing laws and permitting systems, states where there were specific policies targeting cropland fires, and (4) states whose agencies limited the amount of cropland burning in addition to the regulations described above.

## 3. Results

### 3.1. CONUS Trends and Interannual Variability

**Figure 3:**
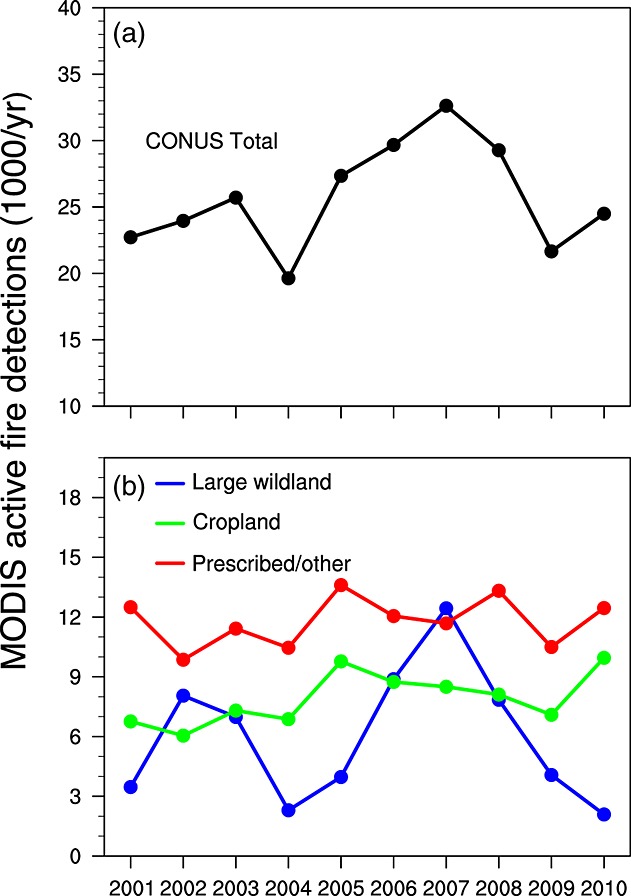
Interannual variations of Terra MODIS active fire detections (number of detections × 10^3^ per year) for (a) the entire U.S., and (b) for large wildland (blue), cropland (green), and prescribed/other (red) fire types. Decadal trends and coefficients of variation are summarized in Table 1.

Large wildland fires accounted for 23% of the total number of active fire detections in the U.S. observed by Terra during 2001–2010. Cropland fires accounted for 31%, and prescribed/other fires for the remaining 46% (Table 1). Active fire detections associated with large wildland fires varied considerably from year to year, with a maximum in 2007 (Figure 3) and a coefficient of variation (CV) that was 3–5 times larger than active fires associated with cropland or prescribed/other fires (Table 1). Active fires in cropland fires increased by 3.4%/yr over the decade (Figure 3, Table 1), whereas active fires associated with large wildland or prescribed/other fires had no significant long-term trends. Overall, cropland and prescribed/other fires were responsible for 77% of the total number of active fire detections in CONUS. Approximately half of those were in the southcentral and southeast regions, where contributions from large wildland fires were relatively small. Analysis of Aqua MODIS data during 2003–2010 showed a similar partitioning of the total number of active fire detections among large wildland (20%), cropland (34%), and prescribed/other classes (46%) (Figure S1). The greater relative contribution of cropland active fires to the CONUS sum for Aqua is consistent with this fire type having a more pronounced mid-day peak in burning [e.g., *Mu et al.*, 2011].

**Table 1:** Regional Statistics for Annual Mean Active Fire Detections, Trends, Average Rate of Change (ROC, %/yr; Defined as Trend Normalized by Number of Mean Active Fires), and Coefficient of Variations (CV, %) for Each of the Three Fire Types^a^

Region/Fire Type	Annual Mean Active Fires (#/yr)	% of Total	Trend (#/yr^2^)	ROC (%/yr)	CV (%)
Contiguous United States					
Large Wildland	6,009	23	−4	−0.1	55.9
Cropland	7,914	31	266	3.4*	16.6
Prescribed/Other	11,783	46	95	0.8	10.6
Total	25,707	100	358	1.4	15.7
Pacific (PA)					
Large Wildland	2,451	57	−40	−1.6	62.4
Cropland	629	15	−37	−5.9**	21.6
Prescribed/Other	1,242	29	−26	−2.1	14.2
Total	4,323	100	−103	−2.4	38.6
Mountain (MO)					
Large Wildland	2,466	65	−90	−3.6	82.3
Cropland	171	4	−7	−3.9	23.1
Prescribed/Other	1,177	31	16	1.4	13.7
Total	3,815	100	−80	−2.1	55.2
Northcentral (NC)					
Large Wildland	206	5	17	8.4	104.1
Cropland	2,628	60	135	5.1*	23.0
Prescribed/Other	1,513	35	45	3.0	32.8
Total	4,348	100	197	4.5	22.9
Northeast (NE)					
Large Wildland	22	3	2	10.8	143.4
Cropland	249	39	−9	−3.7	27.4
Prescribed/Other	369	58	−5	−1.3	21.9
Total	640	100	−12	−1.8	25.7
Southcentral (SC)					
Large Wildland	395	6	29	7.3	54.8
Cropland	1,802	29	80	4.4	28.3
Prescribed/Other	4,104	65	112	2.7*	14.8
Total	6,301	100	221	3.5*	18.0
Southeast (SE)					
Large Wildland	469	7	77	16.5*	90.8
Cropland	2,434	39	105	4.3	24.1
Prescribed/Other	3,377	54	−47	−1.4	16.6
Total	6,280	100	134	2.1	19.7

a Double starred (**) values indicate statistically significant trends at *p* values less than 0.05, and single starred (*) values indicate trends with *p* values less than 0.1.

### 3.2. Spatial-Temporal Distribution of Fire Types

**Figure 4:**
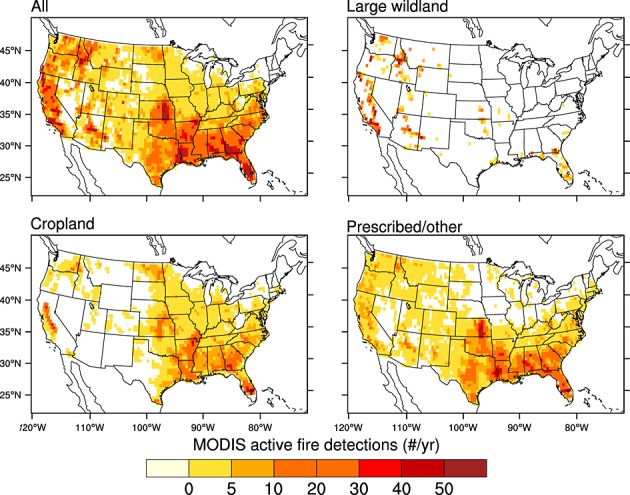
Spatial distribution of annual mean active fire detections across the contiguous U.S. for 2001–2010, for all, large wildland, cropland, and prescribed/other fire types. The attribution process for separating fires into these different classes was performed at a spatial resolution of 1 km and then aggregated to 0.5° grid cells for statistical analysis and mapping. Grid cells that had more than 10 fires during this 10 year interval are shown here.

Each fire type had a unique spatial pattern of active fires across the U.S. (Figure 4). Most large wildland active fires occurred in mountainous regions of the west, with a remainder of 19% distributed across other regions. In contrast, cropland active fires were concentrated within major agricultural regions of the Mississippi Valley, the Central Valley of California, southern Georgia, eastern Washington, Kansas, North Dakota, and Florida. Cropland active fires were relatively rare in the corn belt of the Midwest (Figures 1 and 4), although corn is listed as one of the primary crop types whose residues are regularly burned [*U.S. EPA*, 2010]. Prescribed/other active fires were distributed primarily in south central and southeast regions, across Texas, Louisiana, Mississippi, Alabama, Georgia, and Florida. Other distinctive patterns for this fire type included a large number of active fire detections within the Flint Hills of Kansas and across rangelands in Texas, where prescribed fires in grasslands and on private lands may not be entrained into MTBS data sets.

**Figure 5:**
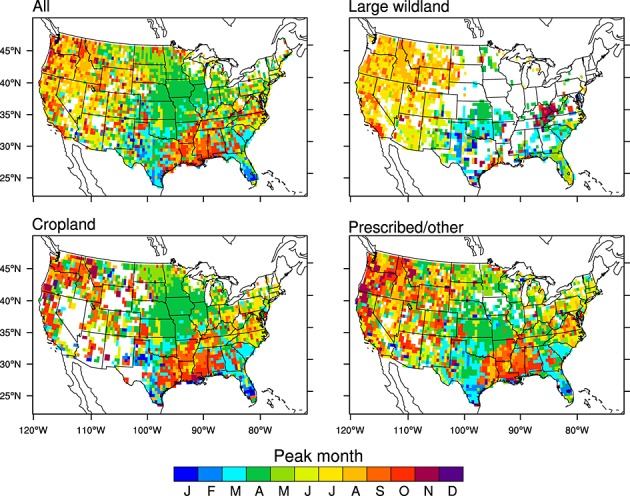
Peak fire month for all, large wildland, cropland, and prescribed/other fire types for the contiguous U.S. aggregated to 0.5°. Regional averages for fire season and peak fire month are shown in Table 2. Only grid cells that had more than one MODIS active fire during 2001–2010 are mapped here.

The timing of peak fire season varied considerably for the three fire types, providing additional evidence in some regions that these were unique classes (Figure 5 and Table 2). Large wildland fires peaked during summer in many areas of the west. In contrast, cropland and prescribed/other fires often had two distinct peaks in spring or early fall. Major cropland areas in west coast states and in the Mississippi Valley, as well as in forested areas in the southeast had periods of maximum burning between August and September. Another peak occurred during February–April in rangelands of Texas and Oklahoma and across croplands in Florida. Additionally, large wildland fires occurred during narrower seasonal windows in most regions, with 46–84% of active fires detected during a consecutive 3 month window. Cropland and prescribed/other active fires were distributed more evenly throughout the year, with approximately 34–73% of cropland and 32–57% of prescribed/other active fires contained within the fire season.

**Table 2:** Peak Fire Season, Fraction of Mean Annual Active Fires in the Fire Season, Correlation Between Active Fires and Potential Evaporation (PE), and Percent Change in Active Fires per Unit of PE During the Fire Season (%/mm)^a^

Region	Fire Season (Month of Year)	% of Active Fires During Fire Season	Number of Active Fires During Fire Season (#/yr)	Correlation Between Active Fires and PE	% Change in Fires per mm of PE
Large Wildland					
Pacific (PA)	7 8 9	80.5	1,973	0.58	1.65
Mountain (MO)	6 7 8	84.4	2,082	0.90	2.41
Northcentral (NC)	3 4 5	55.8	115	0.04	0.23
Northeast (NE)	4 5 6	45.5	10	0.31	1.99
Southcentral (SC)	4 5 6	64.1	253	0.49	1.07
Southeast (SE)	4 5 6	65.5	307	0.75	2.84
Cropland					
Pacific (PA)	8 9 10	44.0	277	0.04	0.05
Mountain (MO)	8 9 10	72.5	124	0.08	0.08
Northcentral (NC)	3 4 5	52.4	1,377	0.05	0.06
Northeast (NE)	6 7 8	42.6	106	0.54	0.47
Southcentral (SC)	8 9 10	44.6	804	0.57	0.65
Southeast (SE)	2 3 4	33.6	819	0.78	1.25
Prescribed/Other					
Pacific (PA)	9 10 11	32.4	402	−0.32	−0.65
Mountain (MO)	8 9 10	42.4	499	0.20	0.28
Northcentral (NC)	3 4 5	57.3	867	−0.08	−0.15
Northeast (NE)	6 7 8	48.2	178	0.65	0.51
Southcentral (SC)	3 4 5	34.5	1,414	0.22	0.20
Southeast (SE)	2 3 4	34.1	1,150	0.53	0.51

a Underlined months indicate the peak fire month.

### 3.3. Interannual Trends by Region

**Figure 6:**
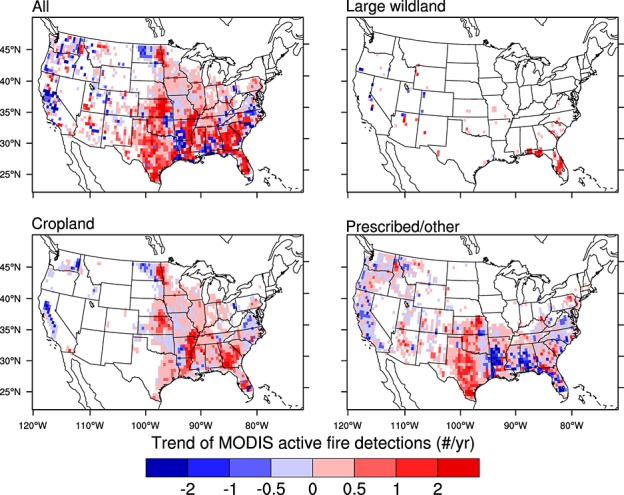
Decadal trends for all, large wildland, cropland, and prescribed/other fire types. Corresponding regional trends and variability are summarized in Table 1.

**Figure 7:**
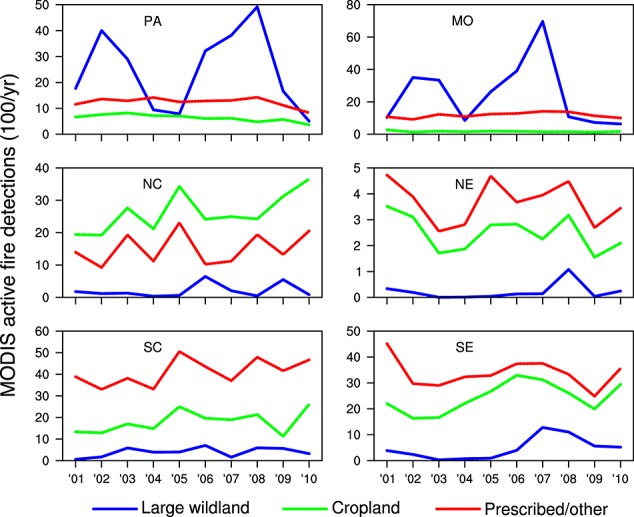
Time series of active fire detections for large wildland (blue), cropland (green), and prescribed/other (red) fire types for the six geographic regions shown in Figure 1. Regional trends and coefficients of variation are summarized in Table 1.

The three fire types contributed in significantly different ways to decadal active fire trends in CONUS (Figure 6). Although the large wildland fire class dominated the overall interannual variation in each region, no significant trends were observed for this class except in the southeast (Figure 7 and Table 1). In this region, active fire detections associated with large wildland fires in Florida contributed to a significant increase (Figure 6).

The long-term increase in cropland active fires in CONUS was driven by contrasting trends in different regions. In the Pacific region, cropland active fire detections decreased significantly (*p* < 0.05) at a rate of 5.9%/yr (Table 1). Decreases were also evident across eastern Washington and western North Dakota (Figure 6). However, at the national scale these negative trends were more than compensated by increases in other areas of the north central, south central, and southeast regions, leading to an overall CONUS increase of 3.4%/yr (Table 1). There was no overall trend for the prescribed/other fire class, with regional increases in Texas offset by decreases in nearby states in the southeast region (Figure 6). At a 0.5° scale, long-term trends for cropland and prescribed/other active fires in individual grid cells often had a different sign (Figure 6). At both regional and continental scales, however, interannual variations of these two fire types were significantly correlated (*r*^2^ values greater than 0.5, *n* = 10 years, *p* values less than 0.05, Figure 7).

### 3.4. Climate Sensitivity

**Figure 8:**
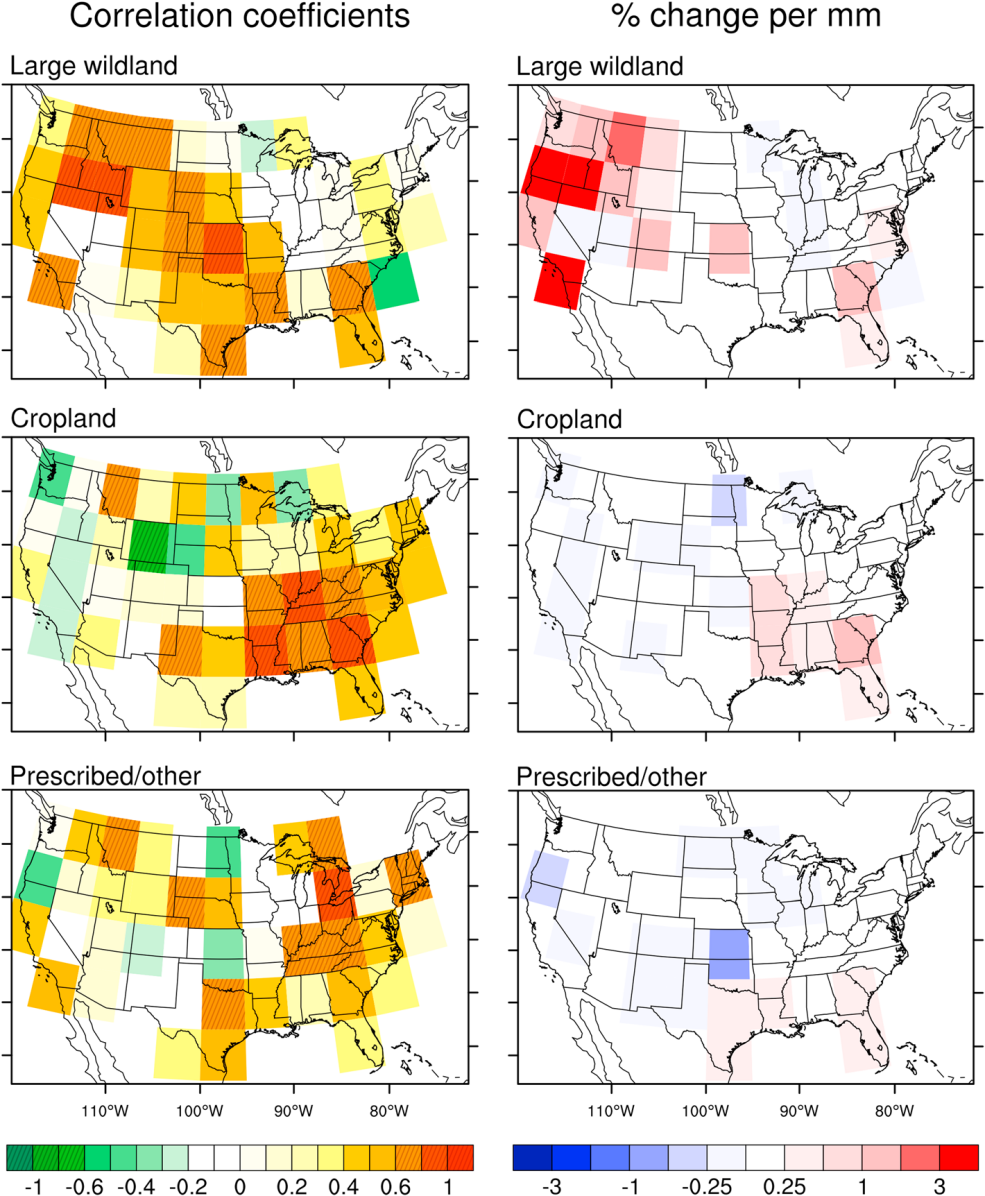
Left column: correlation coefficients between the annual sum of active fire detections and potential evaporation (PE) during the 3 month peak fire season for large wildland, cropland, and prescribed/other fire types. These correlations were calculated at spatial resolution of 5°. Shaded grid cells are statistical significant correlation coefficients (*p* < 0.05, *n* = 10). Right column: relative change in active fires (%) per unit change in PE (mm) during the fire season.

Potential evaporation (PE) during the fire season was a strong regulator of year-to-year variations in the number of active fire detections in the large wildland fire class, with significant positive correlations of 0.58–0.90 in the Pacific, mountain, and southeast regions (Figure 8 and Table 2). Overall patterns of PE control on cropland and prescribed/other fire classes were markedly different than for large wildland fires. In the west, there was no significant relationship between cropland or prescribed/other active fires and PE. In the southeast, in contrast, cropland active fires were significantly correlated with PE at strength comparable to the correlation for the large wildland fire class (Table 2). To estimate the climate sensitivity of active fires in each region to changes in PE, we calculated the percent change in active fires per mm of PE, from a regression of the annual sum of active fires during the fire season and estimated PE during the same period. Using this metric, the climate sensitivities for cropland and prescribed/other fire classes were considerably smaller than for the large wildland fires in most regions (Figure 8 and Table 2). Even in the southeast where the three fire types were significantly correlated with PE, the rate of change for large wildland fires (2.8%/mm) was still more than a factor of two higher than for cropland fires (1.3%/mm) and for prescribed/other fires (0.5%/mm) (Table 2). We also examined the relationship between PE averaged during the 3 months prior to the fire season and the total number of fires during the fire season and did not find statistically significant relationships at a regional scale (results not shown).

### 3.5. Cropland Fire Trends as a Function of the Intensity of Fire Management Policy

**Figure 9:**
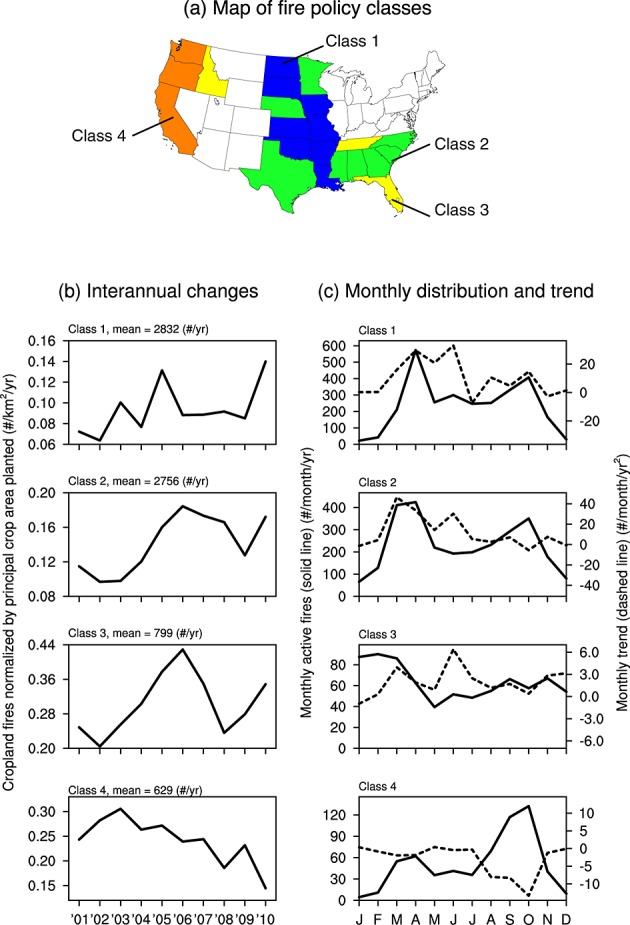
(a) Map of fire management classes identified for different states in CONUS. The intensity of fire management increases from class 1 to class 4. (b) Interannual variations in cropland fires normalized by the principal crop area planted within each management class. (c) Monthly distribution of the active fires within each management class (solid line) and long-term trends (dashed line).

We identified fire regulations and laws in 22 of 48 lower U.S. states, of which we grouped into four fire policy classes based on whether reporting of open burning is required and whether there are specific cropland burning policies (Figure 9a). A description of state-level law articles, regulation systems, and a list of states in each fire policy class are summarized in Table S1. Western states had the highest intensity of fire management policy (class 4), whereas states in other regions often had intermediate levels of intensity (classes 1–3).

In CONUS, 89% of cropland fires occurred in this set of 22 states with fire laws (Figure 9b and Table 1). To account for changes in land use and crop types during our study interval, we quantified interannual changes for cropland fires normalized by principal crop area planted as reported by the U.S. Department of Agriculture (Table S2) for each fire policy class. Fire policy classes 1–3 had considerable year-to-year variability. There was a significant increase in average fire use per square kilometer of principal crop planted for class 2 (*r* = 0.69, *p* < 0.05), which represented 35% of the total cropland fires in CONUS. Class 4, which overlapped with our Pacific region, experienced a significant decrease (*r* = 0.76, *p* < 0.05) and represented 8% of the total U.S. cropland fires. Trends for cropland active fires in classes 1 and 3 did not pass a significance test. The areas covered by fire policy classes 1–4 also accounted for 86% of active fires associated with prescribed/other fires, and for this fire type we found no significant trends among the different fire policy classes (data not shown).

Most of the decrease for class 4 occurred during fall (Figure 9c). In contrast, most of the positive trend for class 2 occurred primarily during spring and early summer. In general, it was difficult to separate influences from multiple agricultural regimes, especially when the fire policy classes spanned discontinuous regions with varying climate and crop types. In the Pacific, however, the seasonal timing of reductions in cropland fires was consistent with efforts to limit fire use during rice harvesting [*California Air Resources Board*, 2008].

## 4. Discussion

### 4.1. Management and Climate Controls on MODIS Active Fire Trends

In this study we assessed the contribution of different fire types to active fire trends in CONUS measured by Terra MODIS during 2001–2010. Our analysis indicated that although the large wildland fire class dominated interannual variability, a much larger number of active fires had a cropland or prescribed/other origin. Our results also provided evidence that in the U.S., the climate sensitivity of fire activity varies with fire type. Comparisons with reanalysis climate data indicated a significant correlation between active fires and potential evaporation for the large wildland fire class, particularly in the west. This finding is consistent with previous studies showing that the number of large wildland fires and total burned area is significantly correlated with potential evaporation and other climate indices in western states [*Westerling et al.*, 2006; *Morton et al.*, 2013]. Climate controls for cropland and prescribed/other fires varied across the CONUS, with no significant correlations between PE and active fires in the Pacific and mountain regions and with positive correlations comparable to those between PE and large wildland fires in the southeast. In the southeast, cropland and prescribed/other fire types also had similar seasonal and interannual patterns (Figures 5 and 7), suggesting that these active fires may respond similarly to climate and management controls.

An important question that emerges from our study is the degree to which intensity of fire management policy influences observed active fire trends for cropland and prescribed/other fire types. We found a significant increase for cropland active fires during the last decade over CONUS with different regional contributions. Active fires in croplands decreased considerably in the Pacific during 2001–2010. This region implemented the most rigorous fire laws after the 1992 Clean Air Act, with the aim of reducing cropland burning to improve air quality. Within this region, Washington and California were the two states that experienced the strongest negative trends in cropland active fires during the last decade. The decrease in the west was more than offset by increases in cropland active fires in other regions where fire use in agricultural management was more prevalent (Table 1 and Figure 6). Fires are widely accepted as a management tool in southeastern states, where water resources and river waste management often are the primary focus of environmental regulations [*Carter and Foster*, 2004]. Kansas, Georgia, and Florida were the three states with the strongest positive long-term trends in cropland fires. Widely distributed positive trends also are visible in Figure 6 for Arkansas and other states encompassing the lower Mississippi Delta Region.

A more robust attribution of fire activity trends to state-level differences in fire policy and management will require further research. Changes in crop production, shifts to crop types that are more fire-intensive, modification of agricultural practices, and land use are likely to be important factors driving observed trends. There was an overall decrease in principal crop area planted in the U.S. across the different fire policy classes in this analysis (Table S2) (data not shown). Taking these decreases in crop area planted into consideration (Figure 9), increases in fires per unit of cropland area may be stronger in many areas than the satellite-derived trends reported here.

Climate appeared to be an important controlling factor on the interannual variability of cropland and prescribed/other fires in the south and southeast (Figure 8). One important unresolved issue is whether this sensitivity reflects change in decisions related to the number of ignitions or indirectly through changes in crop yields or additional management needs due to changes in precipitation and soil moisture. Further work using higher resolution Landsat imagery [e.g., *Masek et al.*, 2006] is needed to resolve this issue. Analysis of Landsat and other high-resolution imagery also is required to improve our understanding of the distribution of prescribed/other fires in relationship to ecosystem type, land management and ownership status, forestry management practices, and cropland areas that were not identified with the coarser resolution MODIS land cover products used here. In the future, higher-resolution land cover and active fire data sets [e.g., *Schroeder et al.*, 2014] will likely enable improved attribution of fires to different land cover types. This has the potential to considerably modify the climate sensitivities for different fire types derived here using moderate-resolution imagery.

### 4.2. Uncertainties and Implications

Satellite observations of active fires are generally more sensitive to fires with a smaller size distribution than many burned area products [*Randerson et al.*, 2012] and therefore may better represent the distribution of fires in cropland, rangelands, and plantations. However, even for the active fire time series analyzed here, it is important to note that many small fires go undetected because they fall below the MODIS detection limit for thermal anomalies [*Morisette et al.*, 2005; *Csiszar et al.*, 2006; *Schroeder et al.*, 2008]. This constitutes an important source of uncertainty for the attribution process developed here and for estimating the contribution of different fire types to emissions. Higher spatial resolution active fire products from the Visible Infrared Imager Radiometer Suite sensor on the recently launched Suomi National Polar-orbiting Partnership satellite [*Coen and Schroeder*, 2013] have potential to considerably improve these estimates in the future.

Our analysis may have several implications for managing fires to reduce impacts on air quality or climate. Current open field burning regulation systems in CONUS span areas that include more than 86% of the active fires associated with cropland and prescribed/other fires types. Since more than 70% of all active fire detections in CONUS during our study period were associated with these two fire types, modification of open field burning policies have the potential to impact regional or continental emissions trends. Evidence for this comes from the contrasting trends for cropland fires in low and high intensity fire policy areas. Cost-benefit and life cycle analyses are needed to assess whether reductions in cropland or prescribed fire emissions—which may improve public health and slow climate warming—would significantly impact crop yields, timber production, soil nutrients, and other ecosystem services and processes.

## 5. Conclusions

In this study, we quantified long-term trends, interannual variability, and seasonality of Terra MODIS active fire detections in the contiguous U.S. as a function of fire type. Large wildland fires had the most variability but no significant long-term trends during 2001–2010. Active fires in croplands decreased by 5.9%/yr in the Pacific, but were more than offset by increases in northcentral, southcentral, and southeast regions, so that the overall CONUS trend was an increase of 3.4%/yr. Potential evaporation exerted a strong control on the interannual variability of active fires associated with large wildland fires and had weaker impacts on cropland and prescribed/other fire types in most regions. Initial analysis indicated that states with more intense (and restrictive) fire management policies had decreasing active fire trends in croplands. By more carefully regulating fire use in land management activities, our analysis suggests opportunities exist to reduce CONUS fire emissions.

## Supporting Information

Readme
