# Supplementary material for: Management and climate contributions to satellite-derived active fire trends in the contiguous United States
Source: J Geophys Res Biogeosci. 2014 Apr 28;119(4):645–60. doi: 10.1002/2013JG002382 (PMC4508926; doi:10.1002/2013JG002382)
Supplement: Supplementary file 1 — Readme [file jgrg0119-0645-SD1.pdf]

Auxiliary material for

Management and climate contributions to satellite-derived active fire trends in the contiguous United States

Hsiao-Wen Lin[1], Jessica L. McCarty[2], Dongdong Wang[3], Brendan M. Rogers[1], Douglas C. Morton[4], G. James Collatz[4], Yufang Jin[1], James T. Randerson[1]

[1]*Department of Earth System Science, University of California, Irvine, California 92697, USA*

[2]*Michigan Tech Research Institute, Ann Arbor, MI 48105*

[3]*Department of Geographical Sciences, University of Maryland, College Park, MD 20742*

[4]*Biospheric Sciences Laboratory, NASA Goddard Space Flight Center, Greenbelt, MD 20771*

Submitted to JGR-Biogeosciences on 13 May, 2013

Revsied on 14 Dec, 2013

The auxilitary infomation contains 2 (two) tables and 1 (one) figure

Table S1. A description of state-level fire law articles and regulations systems and lists of states in each fire policy class used in Figure 9.

Table S2. Principal crop area planted as reported by U.S. Department of Agriculture for each management class. These numbers were used to normalize interannual changes for cropland active fires in Figure 9b.

Figure S1. Time series of Aqua MODIS active fire detections from 2003-2010 for (a) the entire U.S. and for (b) large wildland, cropland, and prescribed/other fire types. This figure is parallel to Figure 3.
