# Supplementary material for: Management and climate contributions to satellite-derived active fire trends in the contiguous United States
Source: J Geophys Res Biogeosci. 2014 Apr 28;119(4):645–60. doi: 10.1002/2013JG002382 (PMC4508926; doi:10.1002/2013JG002382)

Figure S1. Time series of Aqua MODIS active fire detections from 2003-2010 for (a) the entire U.S. and for (b) large wildland, cropland, and prescribed/other fire types. This figure is parallel to Figure 3.

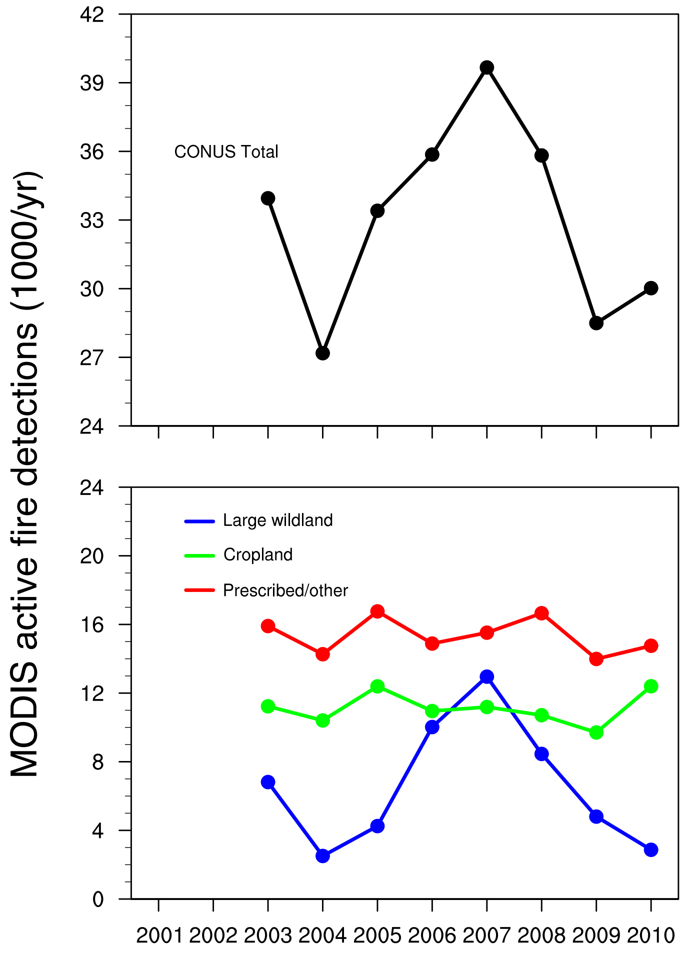

Supplement: Supplementary file 3 — Figure S1 [file jgrg0119-0645-SD3.pdf]
