# Supplementary material for: Management and climate contributions to satellite-derived active fire trends in the contiguous United States
Source: J Geophys Res Biogeosci. 2014 Apr 28;119(4):645–60. doi: 10.1002/2013JG002382 (PMC4508926; doi:10.1002/2013JG002382)
Supplement: Supplementary file 5 — Table S1 [file jgrg0119-0645-SD5.pdf]

Table S1. A description of state-level fire law articles and regulations systems and lists of states in each fire policy class used in Figure 9.

State	State-level fire law (if available)	Description	Does the state use a prescribed burn permit or authorization/notification system?(Y/N)	Are there prescribed burn permit and/or authorization /notification requirements ?	Prescribed burn permit application fee (Y/N)	Does the state offer prescribed burn manager certification courses? (Y/N)	Prescribed fire council	Fire policy class for analysis
**Alabama**[1]	9.13.273	Prescribed burning is considered in the public interest if: 1.) It is accomplished when at least one prescribed burn manager is supervising 2.) A written prescription is prepared and witnessed or notarized prior to burning 3.) A burning permit is obtained 4.) It is conducted pursuant to state law and rule applicable to prescribed burning	Y	Day of burn	N	Y	Established	2
**Arkansas**[2]	Notice to Arkansas Forestry Commission of intent to burn forest vegetation Section 20-22-302	In order to burn forest vegetation, the Arkansas Forest Commission must be notified of intention to burn, time, location, or other relevant information. The landowner or responsible party must be present during the time of burning.	N	--	--	N	Established	1
**California**[3]	Ca. Pub. Res. Code § 4775 (West 1990)	Contracts may be provided for private landowners or a specified public agency by the Director of the California Department of Forestry Burning Contracts in order to prevent high-intensity wild land fires or to achieve other goals (Ex. Forest improvement).	Y	More than 24 hours before burn	Y	N	Established	4
**Florida**[4]	Administrative Code 5I-2.006 Open Burning Allowed	Open burning authorizations require the Certified Prescribed Burn Manager to be on site for the entire burn. The prescription for the burn must be on site and available for inspection by a Department representative.	Y	Day of burn	N	Y	Established	3
**Georgia**	Section 12-6-148	Prescribed burning conducted under the requirements shall be accomplished only when an individual with previous experience is in charge and present on site until the fire is adequately and safely confined.	Y	Day of burn	N	Y	Established	2
**Idaho**	§ 38-405 (1998)	Under the CRB program, growers must obtain approval from DEQ before burning by registering for a Permit-by-Rule at least 30 days before they want to burn. Burn days are limited to weekdays during daylight hours only. Burning on weekends and state and federal holidays is prohibited. Burning can only occur in fields where the crop residue was generated.	Y	More than 24 hours before burn	Y	N	None	3
**Iowa**[5]		It is always preferable to have highly trained firefighters working on a prescribed fire, for both safety and liability reasons. For non-complex, low risk prescribed fire it is recommended that the burn boss be ranked as a Burn Boss 3 or at least have completed the required coursework. For more complex prescribed fires the burn boss should have completed both the coursework and the task book for Burn Boss accreditation. Complex prescribed fires should also have squad bosses who have completed the NWCG Fire Fighter Type 1 training.	N	--	--	N	None	1
**Kansas**	28-19-645. Open	A person shall not cause or permit the open burning of any wastes, structures, vegetation, or any other materials on any premises except as authorized by K.A.R. 28-19-647 and K.A.R. 28-19-648. (Authorized by K.S.A. 1994 Supp.	Y	More than 24	N	N	Established	1

	burning prohibited.	65-3005; implementing K.S.A. 1994 Supp. 65-3005, K.S.A. 65-3010; effective March 1, 1996.)		hours before burn				
**Louisiana**	3:17 (Prescribed burning; intent and purpose; authorization; definitions)	Prescribed burning must: be conducted only under written authority according to the requirements of the commissioner, be conducted only when at least one certified prescribed burn manager is present on site from ignition until the burn is completed and declared safe according to prescribed guidelines, and be considered a property right of the property owner if naturally occurring vegetative fuels are used and when conducted pursuant of prescribed burning laws	Y[12]	Day of burn	--	Y	Established	1
**Minnesota**[6]		All prescribed bum projects on DNR lands, whether contracted or conducted by agency personnel, must be implemented in compliance with a written plan. This plan must have clearly defined and attainable resource management objectives and provide for the protection of public safety including human life, health, and property. Each prescribed bum shall be conducted by qualified personnel.	Y	Day of burn	N	N	None	2
**Mississippi**	Section 49-19-307	Prescribed burning conducted only when at least one (1) certified prescribed burn manager is supervising the burn or burns that are conducted; require that a written prescription be prepared and notarized prior to prescribed burning; require that a burning permit be obtained from the Mississippi Forestry Commission; and be considered in the public interest and shall not constitute a public or private nuisance when conducted pursuant to state air pollution statutes and rules applicable to prescribed burning	Y	Day of burn	N	Y	Established	2
**Missouri**[7]		Agricultural burning is defined in the Missouri Code of State Regulations as "fires set in connection with agricultural or forestry operations related to the growing or harvesting of crops." Unless prohibited by other local laws, regulations or local ordinances, this type of burning is permitted in Missouri.	N	--	--	Y	In Development	1
**Nebraska**[8]		A burning permit from the fire chief with jurisdiction over the area to be burned is required.	Y	Day of burn	N	N	Established	2
**North Carolina**	Section 113-60.40	Prior to conducting a prescribed burning, the landowner shall obtain a prescription for the prescribed burning prepared by a certified prescribed burner and filed with the Division of Forest Resources, Department of Environment and Natural Resources. The prescribed burning shall be conducted in compliance with all the following: the terms and conditions of the open-burning permit, the State's air pollution control statutes, any applicable local ordinances relating to open burning, the voluntary smoke management guidelines, and any rules adopted by the Division of Forest Resources, Department of Environment and Natural Resources.	Y	Day of burn	N	Y	Established	2
**North Dakota**[9]		Although the legislature did not define open burning, the legislative history and common understanding of the term indicates that "open burning" means the burning of any type of combustible material directly into the open air. It includes any outdoor fire, pyrotechnic or flame producing apparatus that has the potential to emit sparks or airborne embers that could present a source of ignition to surrounding outdoor natural or man-made materials. It also includes campfires; charcoal grill or barbeque pit fires; fireworks; garbage pit fires; prescribed burning of prairie, croplands, structures or underbrush in forested areas; outdoor welding operations; burning chemically treated or industrial materials that cannot be easily extinguished; or outdoor fires of any size.	N	--	--	Y	None	1
**Oklahoma**	SECTION 16-28.2. (Prescribed Burning Provisions)	Within 60 days prior to conducting a prescribed burn, the owner of land to be burned shall orally or in writing notify all landowners whose lands adjoin the owner's land to be burned. The owner must also include in the written notice or shall orally notify the adjoining landowners of the proposed date and location of the burn and a telephone number where the owner can be reached for information regarding the prescribed burning. The owner of the land to be burned shall complete the prescribed burning notification plan specified in subsection D of this section and shall submit such plan to the rural fire department nearest the land to be burned.	Y[13]	Day of burn	N	N	Established	1
**Oregon**[10]		Prescribed fire (controlled burn) is used to reduce the risk of large catastrophic wildfires and increase public and firefighter safety, as well as meet a variety of resource management objectives. This planned ignition allows agencies to reduce hazardous fuels and restore habitat and ecosystems. Three general types of prescribed fire are pile burning, understory/under burning, and broadcast burning. Any controlled burning must first get clearance from smoke management officials at the Oregon Department of Forestry or the Washington Department of Natural Resources.	Y	Day of burn	Y	N	None	4
**South**	Statute 48-34-40	Prescribed fires conducted must: have a prescribed fire plan prepared before authorization to burn is given by the State Commission of Forestry, and the	Y	Day of burn	N	Y	Established	2

**Carolina**	Requirements for prescribed fire qualified under this chapter	plan must be on site and followed during the burn; must have at least one certified prescribed fire manager present and supervising the burn from ignition until it is declared safe according to certification guidelines; are considered in the public interest and do not constitute a public nuisance when conducted pursuant to state air pollution statutes, smoke management guidelines, and regulations applicable to the use of prescribed fire; are considered a property right of the property owner						
**South Dakota**[11]		Each prescribed fire conducted by the Division of Wildland Fire Suppression shall have a prescribed fire plan prepared in advance of the planned ignition. These burns shall be done in compliance with an approved plan. All prescribed fires conducted on private lands will have a liability release statement signed by the landowner or his legal designee. Every plan must have definite and measurable resource management or training objectives. There will be no unplanned ignitions.	Y	Day of burn	N	N	None	1
**Tennessee**	Burning on Wildlife Habitat Incentives Program (WHIP) Sites (WHIP 3, 4, 5,	Prescribed burning should be done every two years during January-March to avoid killing new spring grass growth and interfering with nesting season. Fruit and seed production is stimulated while yield and quality increases in herbaceous vegetation and legumes. However, remember to honor the nesting seasons and do not burn during those times.	Y	Day of burn	N	N	Established	3
**Texas**	Section 153.047. Prescribed burning standards	Minimum standards established by the board for prescribed burning must: (1) ensure that prescribed burning is the controlled application of fire to naturally occurring or naturalized vegetative fuels under specified environmental conditions (2) require that at least one certified prescribed burn manager is present on site during the conduct of the prescribed burn;(3) establish appropriate guidelines for size of burning crews (4) include standards for notification to adjacent land owners, the Texas Natural Resource Conservation Commission (5) include minimum insurance requirements for certified prescribed burn managers.	Y[14]	--	--	Y	None	2
**Washington**	RCW 70.94.6528	Outdoor burning is allowed within the urban growth area as described in RCW 70.94.6514 if the burning is not conducted during air quality episodes, or where a determination of impaired air quality has been made as provided in RCW 70.94.473, and the agricultural activities preceded the designation as an urban growth area.	Y	More than 24 hours before burn	Y	N	Established	4

Reference (if available):

1. http://www.forestry.alabama.gov/PB_Act.aspx?bv=1&s=7
2. http://warnell.forestry.uga.edu/fuels/Burning_laws/Arkansas_fire_laws.html
3. http://www.arb.ca.gov/smp/regs/regs.htm
4. http://edis.ifas.ufl.edu/fr055
5. http://www.iowadnr.gov/portals/idnr/uploads/forestry/prescribedfire_guidelines.pdf?tabid=1260
6. http://files.dnr.state.mn.us/forestry/wildfire/rxfire/oporder47.pdf
7. http://www.dnr.mo.gov/pubs/pub2253.pdf
8. http://ianrpubs.unl.edu/epublic/live/ec121/build/ec121.pdf
9. http://www.nd.gov/des/uploads%5Cresources%5C155%5Cburnbanguidelines.pdf
10. http://www.blm.gov/or/resources/fire/prescribedburns/
11. http://sdda.sd.gov/legacydocs/WFS/division/prescribedfire/DOA%20Prescribed%20Fire%20Policy.pdf. South Dakota requires notification by the day of burn to the local fire department. There is an official permitting system in the Black Hills Forest Fire Protection District (http://sdda.sd.gov/wildfire-suppression/burn-permits-faqs). We think Class 1 is most appropriate since only a small fraction of the area within the state has state reporting requirements.
12. Louisiana does not require a permit prior to burn, but burn managers are required to notify local fire agencies before burning.

13. Permits are not required, although a burn plan is encouraged by the Oklahoma Forestry Service.
14. Texas does not require permitting but does require notification via its burn management program to the Texas Commission on Environmental Quality.
