# Supplementary material for: Management and climate contributions to satellite-derived active fire trends in the contiguous United States
Source: J Geophys Res Biogeosci. 2014 Apr 28;119(4):645–60. doi: 10.1002/2013JG002382 (PMC4508926; doi:10.1002/2013JG002382)
Supplement: Supplementary file 7 — Table S2 [file jgrg0119-0645-SD7.pdf]

Table S2. Principal crop area planted as reported by U.S. Department of Agriculture for each management class. These numbers were used to normalize interannual changes for cropland active fires in Figure 9a.

Principal crops area planted (1000 acres)/year	2001	2002	2003	2004	2005	2006	2007	2008	2009	2010
Fire policy class 1	122,692	122,215	123,443	122,808	121,490	120,141	121,599	123,661	122,627	120,220
Fire policy class 2	80,182	81,472	80,341	79,024	77,322	77,477	77,790	77,958	77,827	78,257
Fire policy class 3	10,441	10,641	10,460	10,311	10,056	9,947	10,152	10,305	10,269	10,155
Fire policy class 4	11,028	10,856	10,837	11,015	10,452	10,239	10,204	10,350	9,961	10,137

Principal crops: corn, sorghum, oats, barley, rye, winter wheat, durum wheat, other spring wheat, rice, soybeans, peanuts, sunflower, cotton, dry edible beans, potatoes, sugarbeets, canola, and proso millet. Harvested acreage was used for all hay, tobacco, and sugarcane.

Data source: http://usda.mannlib.cornell.edu/MannUsda/viewDocumentInfo.do?documentID=1000
